# Orthotopic heart transplantation in patient with situs inversus and pectus excavatum: a case report

**DOI:** 10.1186/s40792-024-02006-5

**Published:** 2024-08-30

**Authors:** Satoru Wakasa, Tomonori Ooka, Takuma Sato, Yasushige Shingu, Nobuyasu Kato, Toshiyuki Nagai, Toshihisa Anzai, Minoru Ono, Yoshiro Matsui

**Affiliations:** 1 Department of Cardiovascular Surgery, Faculty of Medicine and Graduate School of Medicine, Hokkaido University, Kita-15, Nishi-7, Kita-Ku, Sapporo, 060-8638 Japan; 2 Department of Cardiovascular Medicine, Faculty of Medicine and Graduate School of Medicine, Hokkaido University, Kita-15, Nishi-7, Kita-Ku, Sapporo, 060-8638 Japan; 3 Department of Cardiac Surgery, Graduate School of Medicine, The University of Tokyo, 7-3-1 Hongo, Bunkyo-Ku, Tokyo, 113-8655 Japan

**Keywords:** Heart transplant, Situs inversus, Pectus excavatum, Chest wall deformity

## Abstract

**Background:**

Heart transplantation in patients with situs inversus is challenging, especially in terms of reconstruction of the systemic venous return. Several rerouting techniques have been presented but are associated with vulnerability to external compression, which might cause hemodynamic instability, especially in the presence of chest deformity. In this study, we report a rare case of successful heart transplantation in the presence of situs inversus and pectus excavatum.

**Case presentation:**

A 55-year-old man, with a history of surgeries for corrected transposition of the great arteries with ventricular septal defect, was registered for heart transplantation owing to progression of heart failure. Subsequently, he had undergone a left ventricular assist device implantation; 14 years after registration, he underwent transplantation of the heart with normal anatomy. The inferior vena cava was reconstructed by anastomosing the left atria with a counterclockwise rotation of the donor heart and by lengthening the recipient inferior vena cava with a conduit made of the residual right atrial tissue. The superior vena cava was reconstructed using a donor innominate vein harvested with sufficient length. After successful weaning from cardiopulmonary bypass, the chest could not be closed because the heart was compressed owing to chest deformity, resulting in hemodynamic instability. Therefore, to exclude the left lung, a left pericardial screen was created using a bovine pericardium, allowing the chest to be closed with acceptable hemodynamics. The patient suffered postoperatively from a higher venous pressure, suggesting an obstruction of venous return early after surgery. The obstruction gradually resolved, and the patient was transferred for rehabilitation.

**Conclusions:**

Heart transplantation in the presence of situs inversus is challenging; moreover, the presence of pectus excavatum further complicates the procedure. The paradoxically larger left lung and chest deformity compressed and impaired reconstructed systemic venous return. Although intrathoracic exclusion of the left lung was effective, an intraoperative or early postoperative thoracoplasty for pectus excavatum was also a viable option. Patient-specific management is mandatory, depending on the anatomy.

## Background

Heart transplantation for patients with situs inversus is challenging [1]. The complex situs arrangements are associated with increased mortality, morbidity, and costs after heart transplantation [2]. Reconstruction of the systemic venous return is the essence of the procedure and can be achieved with several rerouting techniques; however, these techniques are associated with a risk of compression and occlusion [3]. Furthermore, pectus excavatum can severely affect hemodynamics by compressing the transplanted heart [4], especially in combination with the situs inversus. Herein, we report a rare case of successful heart transplantation in the presence of situs inversus and pectus excavatum.

## Case presentation

**Fig. 1:**
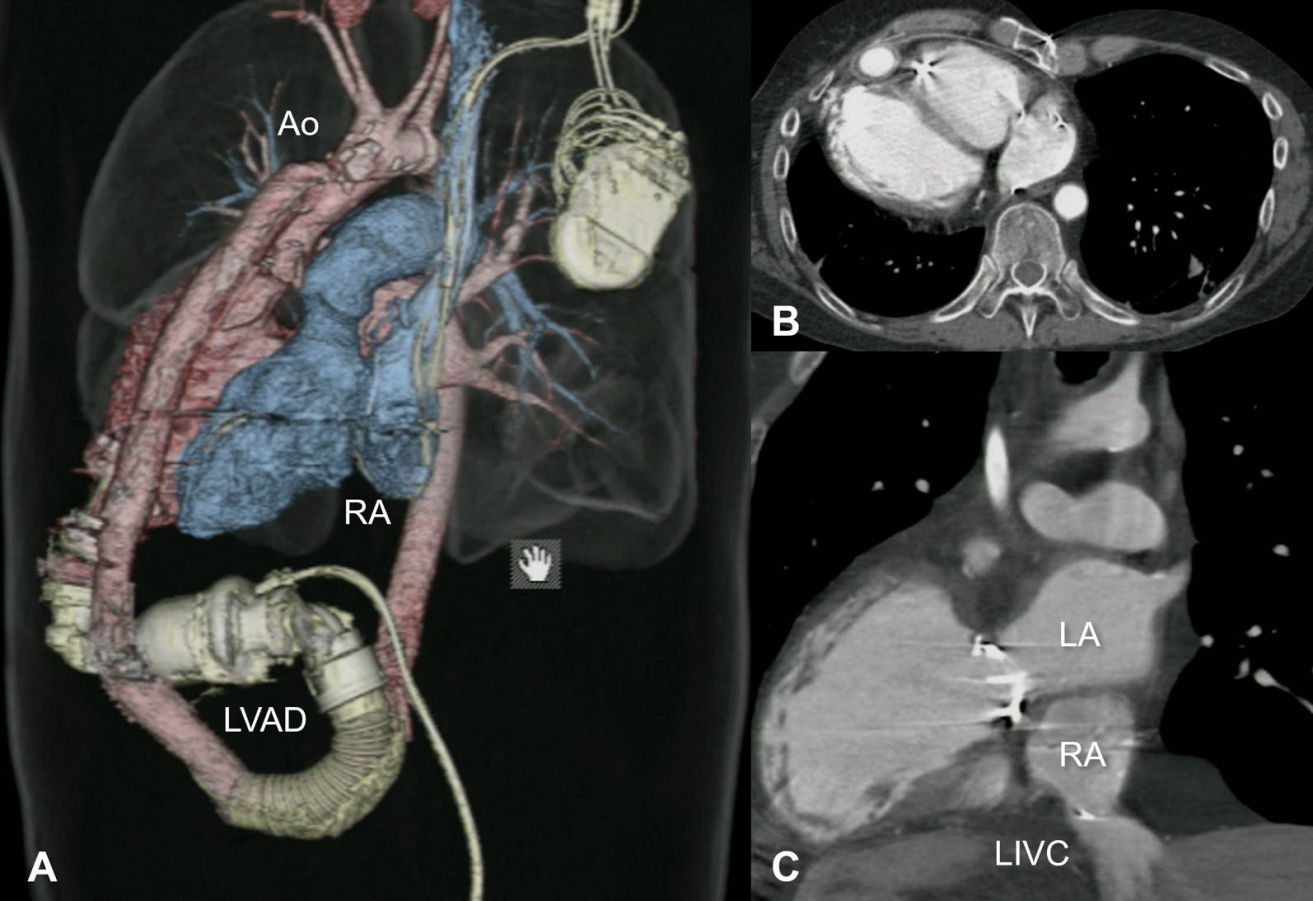
Preoperative computed tomography images: a 3D image showed HeartMate II implanted in a reversed fashion (**A**); an axial image demonstrated a chest deformity (**B**); a coronal image presented the relationship between LA, RA, and LIVC (**C**). *Ao* aorta, *LA* anatomical left atrium, *LIVC* left inferior vena cava, *LVAD* left ventricular assist device, *RA* anatomical right atrium

A 55-year-old Japanese man, born with a corrected transposition of the great arteries with ventricular septal defect, had undergone a closure of the ventricular septal defect at the age of 3, followed by replacement of the anatomical tricuspid valve with a mechanical valve at the age of 33. Thereafter, heart failure had gradually progressed, and he was registered for heart transplantation at the age of 42. Subsequently, he had undergone left ventricular assist device (LVAD) implantation (HeartMate II; Abbott Laboratories, Abbott Park, IL, USA) as a bridge-to-transplantation at the age of 51. Computed tomography revealed the characteristics of the complex anatomy (Fig. 1): the HeartMate II implanted in a reversed fashion with an outflow graft crossing the main body, pectus excavatum with a Haller index of 3.88 [5], left aortic arch, left superior vena cava (SVC) with persistent right SVC, absence of the innominate vein, left inferior vena cava (IVC) with hepatic veins, vertically aligned atria with the caudally located anatomical left atrium, and great arteries in a side-by-side position. The patient presented with renal dysfunction, having an estimated glomerular filtration rate of 42.5 ml/min/1.73 m^2^.

**Fig. 2:**
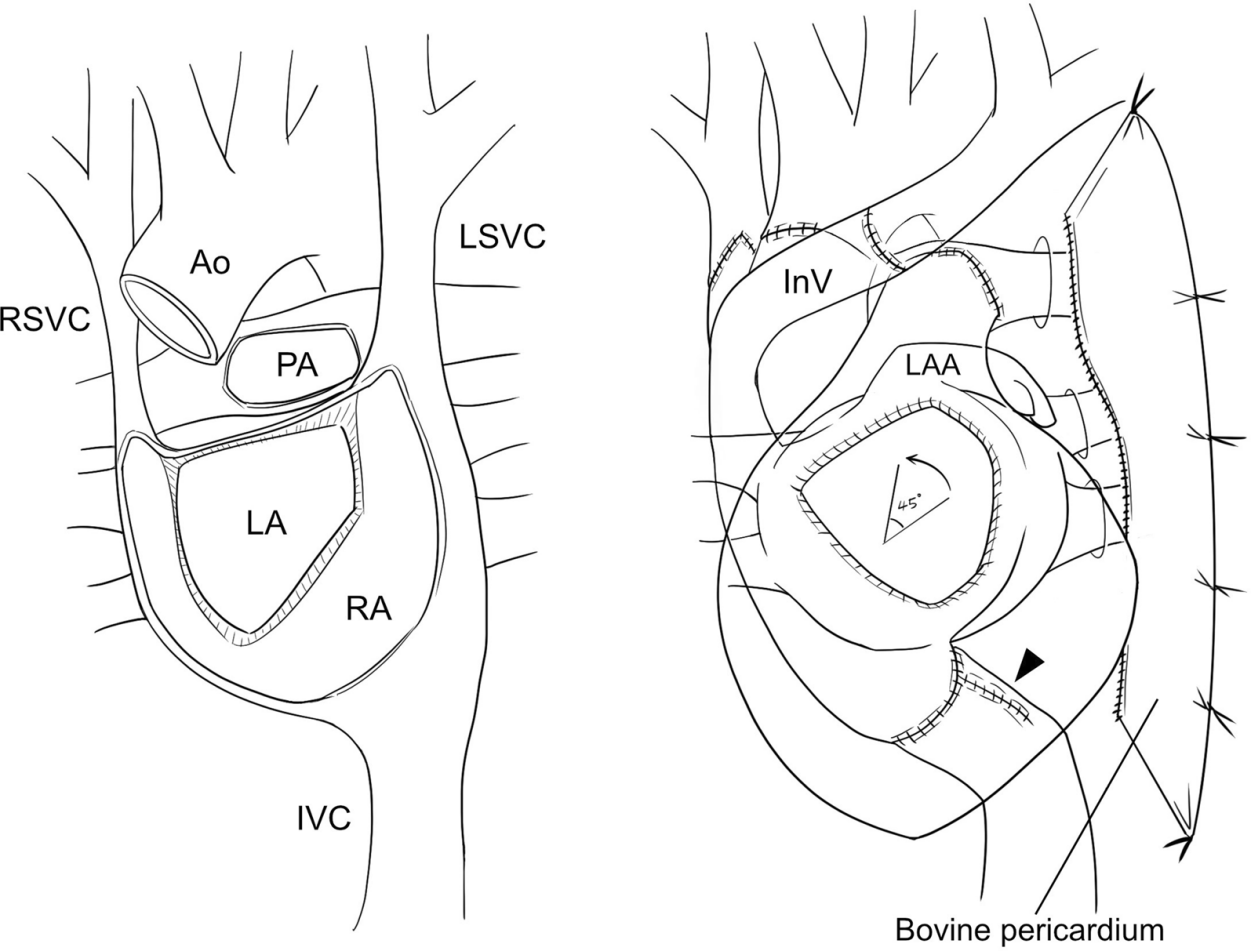
Schematic images after cardiectomy (left) and heart transplantation (right): the left atria were anastomosed with a 45-degree counterclockwise rotation of the donor heart. The recipient IVC was lengthened by a conduit made of residual RA tissues (arrowhead). A bovine pericardium was used to create a screen for excluding the left lung. *Ao* aorta, *InV* innominate vein, *IVC* inferior vena cava, *LA* anatomical left atrium, *LAA* left atrial appendage, *LSVC* left superior vena cava, *PA* pulmonary artery, *RA* anatomical right atrium, *RSVC* right superior vena cava

**Fig. 3:**
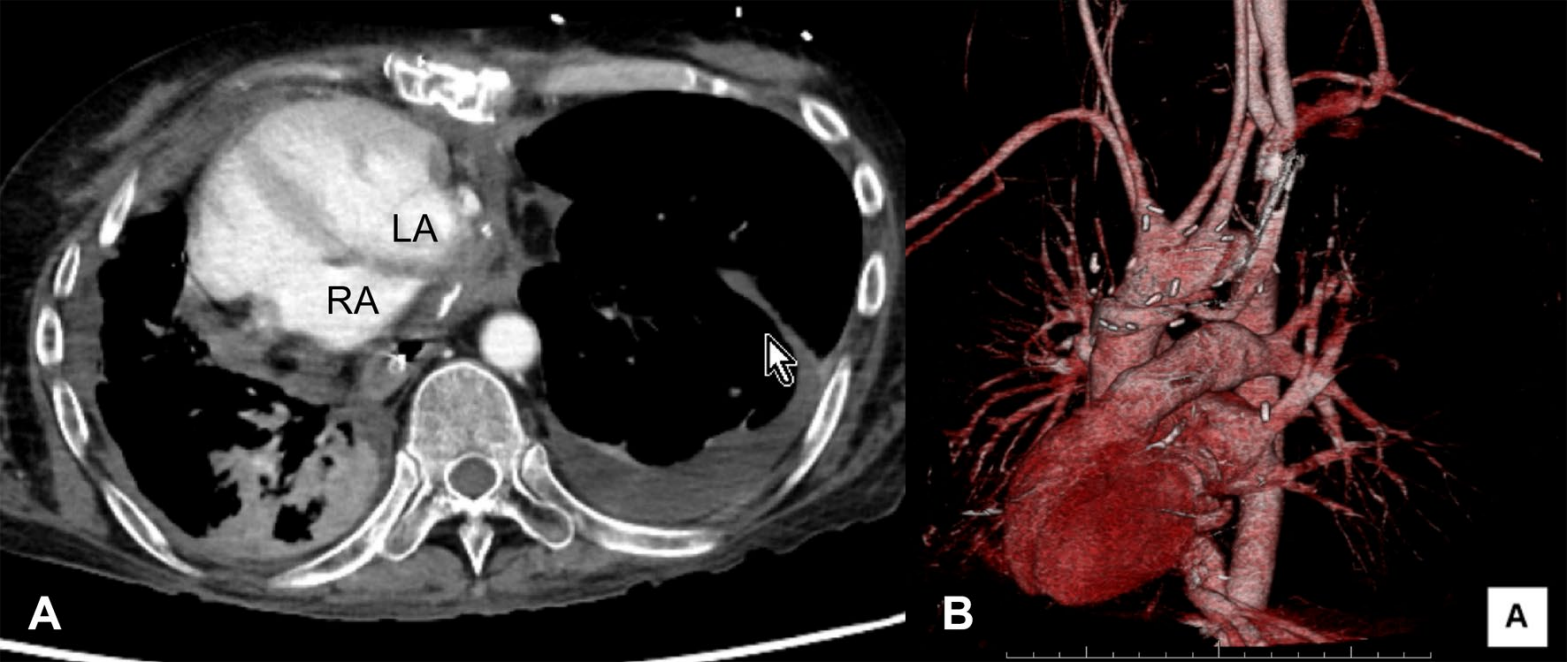
Postoperative computed tomography images: an axial image demonstrated that the transplanted heart was situated in the right hemithorax (**A**); a 3D image showed the heart in a dextrocardia position (**B**). *LA* left atrium, *RA* right atrium

Approximately 14 years after registration (1891 days’ support on LVAD), the patient underwent heart transplantation. The donor was a man in his 40 s with normal cardiac function and anatomy. The donor body surface area was − 9% of the recipient’s size. Procurement of the donor heart was performed as usual. However, to reconstruct the venous drainage from the bilateral SVC of the recipient, the entire length of the SVC and the innominate vein were retained with the donor heart. The recipient operation was performed through a redo sternotomy (Fig. 2). After dissection of the heart and LVAD, cardiopulmonary bypass was commenced with a cannulation on the aortic arch and drainages from the bilateral SVC and IVC. After the aorta was clamped, cardiectomy was performed using a modified bicaval technique. The right atrial tissues were preserved more extensively than usual, especially around the orifice of the IVC. The left atria were anastomosed with a 45-degree counterclockwise rotation of the donor heart to minimize the distance between the donor and recipient IVCs. The remaining right atrial wall around the IVC was trimmed and anastomosed using a 4-0 polypropylene continuous suture to form a conduit with a length of 4–5 cm. Following this, both IVCs were connected using a 4-0 polypropylene continuous suture. After suture of the posterior wall of the pulmonary artery, the ascending aorta was anastomosed. The aorta was declamped, and the suture of the pulmonary artery was completed. The donor right SVC was anastomosed to the recipient narrow right SVC after enlarging its orifice via an additional incision on the anterior wall. The left SVC of the recipient was divided at its connection to the right atrium (RA) and mobilized toward the right side by dividing the azygos vein. The donor innominate vein was then anastomosed to the recipient left SVC. The patient was successfully weaned off cardiopulmonary bypass; however, the chest could not be closed owing to hemodynamic instability. Therefore, a left pericardial screen was created by anastomosing a bovine pericardium posteriorly to the residual autopericardium and anteriorly to the left anterior chest wall apart from the sternum to exclude the left lung and prevent the heart being compressed (Fig. 2, right). The sternum could then be closed with acceptable hemodynamics. The organ transfer and ischemic times were 182 min and 259 min, respectively. No acute rejection was observed. Despite stable hemodynamics, postoperative high venous pressure was observed in the upper body with a pressure gradient of 16 mmHg through the RA to the left SVC, which gradually reduced to 6 mmHg until discharge. Postoperative CT revealed that the transplanted heart was located in the right hemithorax (Fig. 3). During postoperative management, including immunosuppressive therapy, the recipient’s renal dysfunction progressed, and permanent dialysis was required. The patient was transferred to rehabilitation 238 days after the operation.

## Discussion

Reconstruction of the systemic venous return is a significant challenge in heart transplantation in patients with situs inversus. Two main procedures are carried out for reconstruction of the IVC: extension of the recipient IVC and approximation of the recipient and donor IVCs. The former procedure uses pericardium, diaphragm, and residual right atrial tissues [1, 3, 6], while the latter is achieved by anastomosing the left atria with a counterclockwise rotation of the donor heart [7]. In our case, IVC reconstruction was successfully achieved by using both procedures because the donor IVC was expected to be located further from the recipient IVC owing to the caudal location of the recipient left atrium. In the reconstruction of the venous return from the upper body, interposition is commonly performed between the recipient and donor SVCs using the innominate vein and a prosthetic graft [1, 3, 6]. In our case, we used the donor innominate vein, a sufficient length of which could be harvested to bypass the left SVC of the recipient. The division of the recipient azygos vein was also effective in mobilizing the SVC, despite the possible adverse effect of eliminating the azygos connection.

Pectus excavatum is rarely encountered in patients undergoing heart transplantation and may disturb the procedure and cardiopulmonary function [8]. Jaroszewski et al. reported two successful cases of pectus excavatum repair [4]. Because the sternum could not be closed, the repair was performed simultaneously and 3 days after transplantation. In our case, the stabilization of hemodynamics after excluding the left lung suggested that the left lung, which was paradoxically larger than the right one after the transplantation, predominantly affected the hemodynamics by expanding within the deformed chest after mechanical ventilation resumed. Repair of chest wall deformity was also considered intraoperatively but abandoned owing to the elongated operation time, thin rather than concave chest shape, and acceptable hemodynamics after chest closure with left lung exclusion. However, an intraoperative or early postoperative thoracoplasty would be required in cases with more concave chest deformity. Because perioperative management strategies have not been established owing to the rarity of the situation, further study is needed to elucidate the ideal management for concomitant chest deformity in heart transplantation, especially for patients with situs inversus.

## Conclusions

The transplanted heart in the situs inversus chest was susceptible to the compression caused by the paradoxically larger left lung, especially in the presence of the chest deformity. Patient-specific management is required, including exclusion of the left lung and thoracoplasty.

## Data Availability

The data supporting the findings of this study are available from the corresponding author upon reasonable request.

## Abbreviations

IVC: Inferior vena cava
LVAD: Left ventricular assist device
SVC: Superior vena cava
RA: Right atrium
